# Nanotechnology in Osteogenesis and Inflammation Management: Metal–Organic Frameworks, Metal Complexes, and Biomaterials for Bone Restoration

**DOI:** 10.3390/biomedicines13071597

**Published:** 2025-06-30

**Authors:** Bogdan Huzum, Ionut Iulian Lungu, Ovidiu Alexa, Paul Dan Sirbu, Viorel Dan Cionca, Andreia Corciova, Andreea Lungu, Monica Hancianu, Ionela Lacramioara Serban, Oana Cioanca

**Affiliations:** 1 Faculty of Medicine, “Grigore T. Popa” University of Medicine and Pharmacy, Independentei Street, No. 16, 700115 Iasi, Romania; 2 “St. Spiridon” County Clinical Emergency Hospital, Independentei Bd., No. 1, 700111 Iasi, Romania; 3 Faculty of Pharmacy, “Grigore T. Popa”, University of Medicine and Pharmacy Independentei Street, No. 16, 700115 Iasi, Romaniaoana.cioanca@umfiasi.ro (O.C.)

**Keywords:** osteoporosis, osteogenesis, flavonoids, nanotechnology, implants

## Abstract

A varied family of polyphenolic chemicals, flavonoids, are becoming more and more important in bone tissue engineering because of their osteogenic, anti-inflammatory, and antioxidant effects. Recent developments incorporating flavonoids into different biomaterial platforms to improve bone regeneration are emphasized in this study. Osteocalcin (OCN) expression was 2.1-fold greater in scaffolds loaded with flavonoids—such as those made of polycaprolactone (PCL)—greatly increasing human mesenchymal stem cell (hMSC) proliferation and mineralization. Comparably, a threefold increase in calcium deposition indicates increased mineralization when hydroxyapatite (HA) was functionalized with flavonoids such as quercetin. These HA scaffolds with flavonoids also showed a 45% decrease in osteoclast activity, therefore promoting balanced bone remodeling. Concurrent with flavonoids like EGCG and quercetin, chitosan-based scaffolds encouraged osteogenic differentiation with increases in osteogenic markers like osteopontin (OPN) and alkaline phosphatase (ALP) expression by up to 82%. These scaffolds also showed 82% bone defect repair after six weeks in vivo, suggesting their promise in rapid bone regeneration. With an increase of up to 32% in the bone volume-to-total volume ratio (BV/TV) and 28% greater bone–implant contact (BIC), flavonoid coatings on titanium implants enhanced osteointegration in implantology. Displaying successful osteogenesis and immunomodulation, the addition of flavonoids into metal–organic frameworks (MOFs) and injectable hydrogels demonstrated a 72% increase in new bone formation in vivo. Though further research is required to confirm long-term clinical effectiveness, these findings show the great promise of flavonoid-functionalized biomaterials in bone regeneration.

## 1. Introduction

From acute fractures to chronic diseases like osteoporosis, bone injuries present a great worldwide health concern with increasing frequency and intensity. The Global Burden of Disease Study 2019 estimates that, compared to 1990, there were around 178 million new fractures globally that year—a startling 33.4% increase. The projected 25.8 million years lived with disability result from the estimated 455 million persons living with a fracture-related disability at the same time. With most usually resulting from osteoporosis, fragility fractures account for a large fraction of these injuries and disproportionately impact women and older persons [1,2,3,4,5].

Affecting more than 500 million individuals globally, osteoporosis by itself is a quiet pandemic. Every three seconds, there is one osteoporotic fracture out of more than 8.9 million fractures reported yearly. Within the European Union, fractures linked to osteoporosis are estimated to cost the healthcare system EUR 37 billion annually. Direct healthcare expenses linked to osteoporotic fractures in the United States topped USD 52 billion in 2020 and are expected to increase significantly with population aging. Over an individual’s lifespan, over one in two women and up to one in four males over the age of 50 may have an osteoporosis-related fracture [6,7,8,9,10,11,12].

These alarming figures highlight how urgently more efficient approaches for bone repair and regeneration are needed. Adverse effects of current conventional treatments—bisphosphonates, selective estrogen receptor modulators, parathyroid hormone analogues, and monoclonal antibodies— include gastrointestinal disorders, the risk of atypical fractures, and osteonecrosis of the jaw. Furthermore, while helpful for acute injuries, surgical treatments are intrusive and not usually successful in older or osteoporotic individuals because of poor healing responses [13,14,15,16,17].

In recent decades, regenerative procedures have garnered significant attention due to the increasing demand for effective strategies to restore and regenerate lost or damaged bone tissue. Current clinical approaches typically involve the use of autologous bone grafts, allografts, xenografts, and platelet concentrates—such as platelet-rich plasma and platelet-rich fibrin—which aim to enhance the biological environment for bone healing. These methods are often combined with bioactive scaffolds or osteoinductive agents to improve therapeutic outcomes [18,19,20]. Experimental strategies continue to evolve, focusing on advanced biomaterials including MOFs, flavonoid–metal ion complexes, and hybrid coatings, which can modulate bone cell behavior, promote angiogenesis, and control local drug delivery. Such combinatory systems are increasingly investigated for their potential to synergistically support bone regeneration, particularly in osteoporotic contexts where bone remodeling is compromised [21,22,23].

Polyphenols, a broad class of plant secondary metabolites recognized for their antioxidant, anti-inflammatory, and signaling-modulating powers, are naturally occurring substances with osteoprotective effects that have attracted more attention recently. Among these, flavonoids—containing subclasses like flavonols, flavanones, isoflavones, and anthocyanidins—have emerged as especially promising because of their broad bioactivity range and availability in regularly eaten foods such berries, onions, apples, green tea, and citrus fruits [24,25,26,27,28,29,30].

Flavonoids have many positive impacts on bone tissue by means of several processes. While simultaneously reducing the differentiation and resorptive activity of osteoclasts (bone-resorbing cells), at the cellular level flavonoids increase the activity, differentiation, and survival of osteoblasts (bone-forming cells) [31,32,33]. They alter important processes in bone remodeling, including the following:

- Essential for osteoblast proliferation and maturation, Wnt/β-catenin signaling [34,35,36].
- Pathways of BMP/TGF-β control of mesenchymal stem cell development into osteoblasts [37,38,39].
- MAPK pathways, affecting gene transcription related to bone development [40,41].
- Central control of osteoclastogenesis and bone resorption: RANK/RANKL/OPG signaling [42,43].

Moreover, flavonoids are strong antioxidants that neutralize reactive oxygen species (ROS), which are recognized to induce osteoclast development and compromise osteoblast activity. Flavonoids maintain a favorable redox state fit for bone anabolism by lowering oxidative stress. Strong anti-inflammatory actions also help to reduce the production of pro-inflammatory cytokines (e.g., TNF-α, IL-1β, IL-6), which are typically raised in chronic bone diseases and fracture healing delay [44,45,46,47].

Despite these encouraging bioactivities, low oral bioavailability, poor stability, and fast metabolism have hampered the therapeutic application of flavonoids in bone repair. Researchers have started looking at the utilization of flavonoid–metal complexes, a novel approach using coordination chemistry to improve the pharmacokinetics and pharmacodynamics of flavonoids in order to overcome these obstacles [48,49,50,51].

Flavonoids are ligands in these complexes chelating physiologically relevant metal ions including calcium (Ca^2+^), zinc (Zn^2+^), magnesium (Mg^2+^), iron (Fe^3+^), and copper (Cu^2+^). Apart from their essential role in enzyme activity and bone mineralization, these metal ions provide the flavonoid complexes with extra biological action. Metal coordination may boost flavonoids’ cellular absorption, protect them from metabolic breakdown, and increase their structural stability [52,53,54].

Research on flavonoid–metal complexes reveals better osteogenic, antioxidant, and anti-inflammatory effects than those of pure flavonoids. For example, quercetin–zinc [55,56] and hesperetin and its derivatives [57,58,59] have been shown to increase ALP activity, run-through osteogenic gene expression (e.g., Runx2, OCN), and hasten matrix mineralization in vitro. In osteoporotic animals, in vivo, these complexes have shown promise in increasing bone density, accelerating fracture healing, and reversing bone loss.

The clinical potential of flavonoids in regenerative biomaterials is greatly limited by their poor aqueous solubility and low systemic bioavailability. For instance, baicalein exhibits solubility in the range of 17–90 µg/mL in water at room temperature, while formulations such as phospholipid complexes or glycyrrhizic acid-based nano-micelles show increased solubility of over 1500 µg/mL and even more than 4,500-fold, respectively. Quercetin and myricetin, both classified as BCS Class II compounds, typically dissolve at less than 2 µg/mL in water and show oral bioavailability below 1%. Rutin, a glycosylated form of quercetin, shows slightly better water solubility (~125 µg/mL), but remains poorly absorbed in vivo. Luteolin also has limited solubility (<10 µg/mL) and undergoes extensive first-pass metabolism. Even catechin, while relatively more soluble (~125 µg/mL), presents challenges for sustained delivery due to its instability in physiological conditions. Despite these drawbacks, flavonoids such as quercetin, catechin, baicalein, luteolin, myricetin, and rutin have demonstrated significant bioactivity relevant to tissue regeneration. To overcome solubility-related limitations, recent research has focused on the development of metal–flavonoid complexes, nano-micelles, cocrystals, and polymeric dispersions. These delivery systems improve solubility, bioavailability, and therapeutic efficacy, enabling sustained and localized release. Such strategies are particularly relevant in bone regeneration and wound healing under impaired conditions like diabetes and osteoporosis, where oxidative stress and poor vascularization hinder healing [60,61,62,63,64,65].

Focusing on both osteoporosis and acute bone damage, this study attempts to fully investigate the present scene of flavonoid–metal complexes as developing treatments in bone regeneration. It starts with a review of the pathophysiology and epidemiology of bone injuries, then goes into great depth on the ways in which flavonoids enhance bone health.

Finally, it explores the synthesis, characterization, and biological assessment of flavonoid–metal complexes, stressing their potential to transform the discipline of bone regeneration medicine.

## 2. Materials and Methods

The purpose of this extensive literature search was to identify relevant studies that investigated the biomedical applications of flavonoids, such as quercitrin, icariin, rutin, and kaempherol, particularly in the context of metal complexes, surface coatings for bone implants, and MOFs, with a particular emphasis on the osteogenic and anti-osteoporotic properties of flavonoids. With the help of the appropriate Boolean operators (AND/OR), searches were conducted in the databases Google Scholar, PubMed, and ScienceDirect. The controlled vocabulary and free-text terms that were used were as follows: “flavonoids”, “osteogenesis”, “osteoblast proliferation”, “bone regeneration”, “coated implants”, and “osteoporosis.”

In addition, the reference lists of the publications that were chosen were looked through manually in order to locate other studies that were pertinent. Through the utilization of Zotero (version 7.0, Corporation for Digital Scholarship, Fairfax, VA, USA), duplicate records were eradicated.

For the purpose of determining eligibility, full-text papers were evaluated by two independent reviewers. Disagreements were settled by collaborative conversation, and if required, a third reviewer was brought in to make a decision. Eligible studies included interventional research that examined the effects of flavonoid-based materials or derivatives in promoting osteogenesis or treating bone-related illnesses, compared to appropriate control groups. These studies were conducted in vitro or in vivo. Articles, case reports, reviews, and opinion pieces written in languages other than English were excluded.

The process of data extraction was carried out separately by two investigators utilizing a standardized form. The data that were extracted included the first author, the year of publication, the kind of research, the experimental model, the type of intervention (for example, flavonoid–metal complex, implant coating, or MOF structure), the sample size, and the results that were either osteogenic or therapeutic. In order to give a thorough and integrated assessment of the present scientific landscape, studies that used cellular models, animal models, and pertinent preclinical trials were included in the review.

## 3. Synergistic Flavonoid–Metal Combinations: An Approach

The strong antioxidant qualities of flavonoids are very important in reducing oxidative stress in bone illnesses including osteoporosis. By upsetting bone homeostasis, lowering osteoblast differentiation, and encouraging osteoclastogenesis, oxidative stress is clearly a major factor causing bone degradation [66,67,68]. Many studies have shown that flavonoids may reduce these effects and encourage bone development in combination with metal ions (Table 1).

For its antioxidant and osteogenic properties, for instance, quercetin has been extensively examined. Quercetin-loaded microspheres created by Han et al. (2022) [73] caused macrophage polarization to move toward the M2 phenotype. Since M2 macrophages generate anti-inflammatory cytokines that encourage bone mending, this change helps tissue restoration. Moreover, these microspheres improve ALP activity—a sign of osteoblastic differentiation.

**Figure 1:**
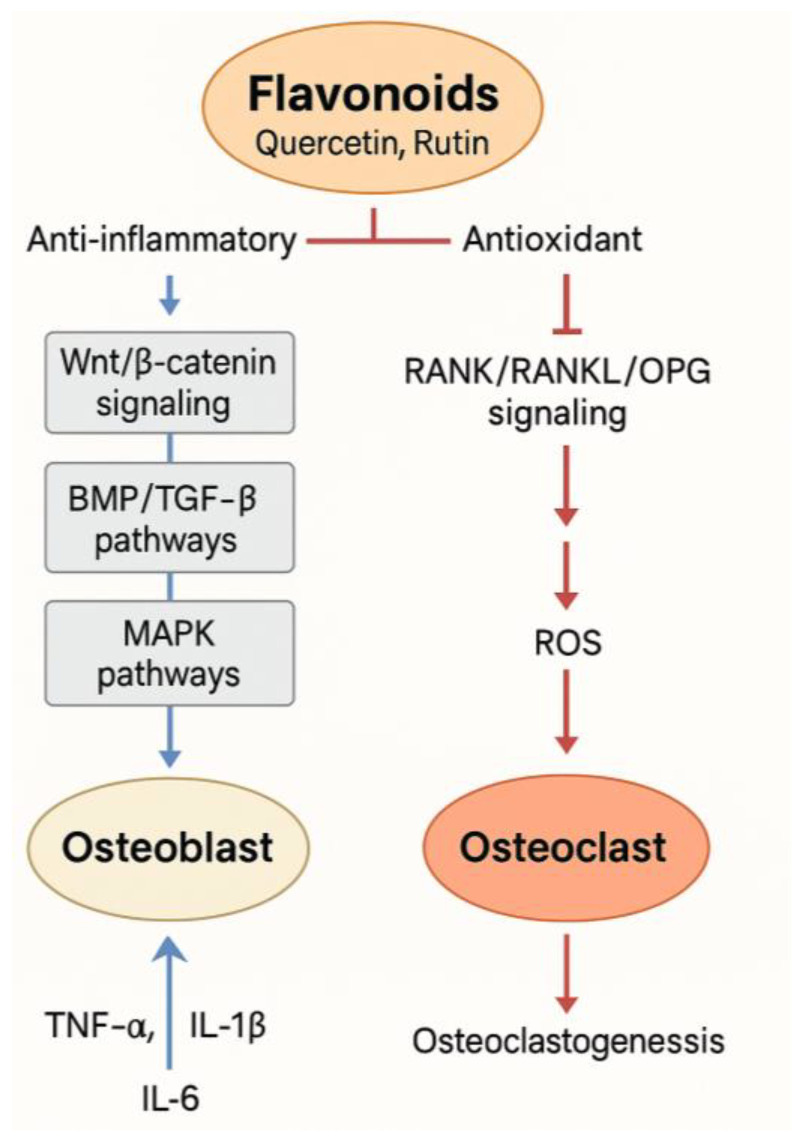
Mechanisms of flavonoids in bone remodeling.

Another flavonoid that has notable anti-inflammatory and osteogenic properties is dihydromyricetin. Yang et al. (2024) [74] incorporated dihydromyricetin into hydrogels to increase osteoblast activity and lower pro-inflammatory cytokines like TNF-α. Modulating the PI3K and MAPK signaling pathways, which are important in cell survival, differentiation, and immune control, helped to explain this dual effect of osteogenesis and anti-inflammation (Figure 1).

The antioxidant-based flavonoid rutin has been found to control extracellular matrix (ECM) protein production. Rutin enhances OPN and ALP expression, therefore supporting osteoblast activity. Rutin also reduces p53 activity, a protein in charge of controlling cell cycle progression and death [75]. This activity implies that rutin not only increases osteogenesis but also stops too much cell death, hence preserving bone homeostasis.

### 3.1. Copper and Flavonoid Complexes

Copper greatly affects the processes of collagen cross-linking, angiogenesis, and osteogenesis. The development of mixed-ligand copper(II)–quercetin complexes has clear negative consequences for biological systems. In human mesenchymal stem cells (hMSCs), Vimalraj et al. (2018) [48] showed that these complexes greatly encouraged both angiogenic and osteogenic development. The complexes increased ALP activity by forty-five percent and vascular endothelial growth factor (VEGF) expression by sixty percent. The findings of their research show that when combined with flavonoids, copper significantly increases osteogenic functioning.

Other studies, including the one carried out by Rajalakshmi et al. (2018) [72], further validate these findings and offer additional proof of their validity by combining silibinin/phenanthroline/neocuproine copper complexes. ALP and OCN were two of the markers that these molecules greatly raised. These drugs also produced a 1.6-fold boost in osteoblast growth. Furthermore, indicating increased mineralization potential, Gaddi et al. (2023) [51] found that copper–flavonoid complexes raised ALP activity by more than 70%. The main processes by which these complexes exert their effects include activation of the ERK1/2 and PI3K/Akt signaling pathways, upregulation of osteogenic genes including VEGF and RunX2, and effective scavenging of ROS.

### 3.2. Zinc and Flavonoid Complexes

Apart from being a necessary trace element for bone development, zinc is particularly well known for its function in the mineralization of compounds and enzyme activation. The incorporation of zinc complexes into polymer matrices yields results that show promise. Preeth et al. (2021) [56] saw an 83% increase in calcium deposition by incorporating a Zn(II) complex into PCL/gelatine nanofibers above the control scaffolds. This finding especially emphasizes the osteoconductive role that zinc plays in a biomimetic setting.

More recently, Vimalraj et al. (2019) [70] showed that zinc–morin complexes raised osteogenic markers more significantly than morin alone. The study team confirmed this. Kaempferol–zinc complexes significantly raised vertebral mineralization, according to in vivo studies performed using zebrafish models. These results further support the possibility of translating these compounds into other species. The Wnt/β-catenin signaling pathway, collagen type I alpha 1 (COL1A1), and changes in bone morphogenetic protein-2 (BMP-2) mechanistically create these effects [69].

### 3.3. Additional Metal Complexes

Flavonoid–metal synergy offers benefits not just with one kind of ion. Rutin–Zn(II) complexes have been shown, according to Vimalraj et al. (2021) [71], to induce osteogenic gene expression in human dental pulp stem cells (hDPSCs) and to help mineral nodules develop. Selvaraj et al. (2021) [76] likewise found that ferulic acid combines with copper(II) and zinc(II) to enhance osteogenesis by means of MAPK signaling.

Moreover, Fernández-Villa et al. (2024) [77] demonstrated that creative systems, such nanocomplexes made of europium and tannic acid, have the capacity to preserve osteogenic potential even when exposed to oxidative stress, a main condition that usually impedes bone regeneration. Taken together, all of these results highlight the fact that various flavonoid–metal combinations have the ability to affect the behavior of cells and therefore encourage bone regeneration in a variety of physiological contexts.

### 3.4. Biomaterials Made from Flavonoid Electrospun Nanofibers

Emulating the fibrous structure of the ECM, electrospinning is a flexible and effective technique for creating nanofibrous scaffolds with high surface-area-to-volume ratios. Because this method can provide an appropriate milieu for cell adhesion, proliferation, and differentiation, it has become somewhat well known in bone tissue engineering. Including flavonoids into electrospun nanofibers has various benefits, including the persistent release of bioactive molecules that may over time constantly activate osteogenic pathways, therefore fostering bone repair [78,79,80,81].

In their study, Raja et al. (2021) [82] incorporated polyphenols—including flavonoids—into electrospun nanofibers produced from biocompatible materials like PCL. hMSC proliferation and mineralization were shown to be much improved by these nanofibers. This system’s continuous release of flavonoids most certainly helped to upregulate important osteogenic markers, hence improving the mineralization process. This suggests that electrospun nanofibers are not only useful for structural support but also a dynamic mechanism for flavonoid delivery to improve bone development.

### 3.5. Functionally Modified HA

Because HA somewhat closely resembles the mineralized component of real bone, it is among the most often employed materials in bone regeneration. Still, HA in its pure form does not always have enough bioactivity to activate the complicated mechanisms of bone mending. By means of flavonoids, functionalizing HA improves its bioactivity, thereby offering two advantages: it not only preserves its osteoconductive qualities but also increases its osteoinductive ability [83,84,85,86,87].

The work by Forte et al. (2016) [88], in which quercetin-functionalized HA scaffolds were employed to enhance osteoblast–osteoclast–endothelial co-culture, is a prime illustration of this method. The threefold increase in calcium deposition as a result of the modified HA scaffolds over unmodified HA shows how much the flavonoid-functionalized scaffolds greatly promote mineralization. This is very important for bone regeneration as the creation of mechanically competent bone depends on appropriate mineralization. Moreover, the co-culture paradigm used in the work showed that quercetin-functionalized HA scaffolds may concurrently control osteoclast and osteoblast activity. A hallmark of osteoblast differentiation, quercetin was demonstrated to downregulate RANKL, a fundamental component involved in osteoclastogenesis, and upregulate OCN. These findings imply that HA scaffolds with flavonoid-functionalizing properties may efficiently balance osteogenesis and osteoclastogenesis, thereby assuring that bone development progresses in harmony with the resorption process.

The usage of quercetin-functionalized HA also emphasizes the multifarious function of flavonoids in modifying the bone remodeling process, indicating that these scaffolds may be especially helpful in circumstances where osteoclast overactivity, like as osteoporosis, needs to be reduced. Flavonoid-functionalized HA scaffolds provide a potent weapon for focused treatments meant to increase bone regeneration and stop overly strong bone resorption by changing the balance between osteoclasts and osteoblasts.

### 3.6. Chitosan-Based Systems

The excellent biocompatibility, biodegradability, and capacity to create hydrogels make chitosan—a biopolymer derived from chitin—an attractive material for use in bone tissue engineering. Combining chitosan-based scaffolds with flavonoids may provide efficient platforms for regulated drug release, therefore enabling the continuous administration of bioactive molecules that improve osteogenesis and reduce inflammation. Chitosan’s hydrophilic nature helps it to absorb and release significant quantities of bioactive substances, therefore influencing cellular responses in bone repair [89,90,91,92].

In 2024, Yang et al. [93] showed how well chitosan scaffolds loaded with zinc–epigallocatechin gallate (Zn-EGCG) promoted osteogenesis and angiogenesis. These scaffolds produced greater osteogenic differentiation and greater vascularization in in vitro experiments. This is important as the survival of recently developed bone tissue depends on angiogenesis. These scaffolds enabled 82% defect closure in six weeks when implanted in rat models, an amazing result suggesting their possible speed and efficiency in supporting in vivo bone regeneration.

Li et al. (2024) [94] investigated quercetin–chitosan conjugates, demonstrating that these scaffolds dramatically elevated OPN and ALP gene expression—both of which are important indicators of osteogenic differentiation. This further highlights the part that flavonoids play in encouraging osteoblast activity and bone matrix production. Furthermore, the coupling of chitosan with flavonoids seems to improve the mechanical characteristics of the scaffold as the polymeric structure offers the required support while the flavonoids control cellular reactions.

Apart from encouraging osteogenesis, flavonoids incorporated into chitosan scaffolds also help to lower oxidative stress and the production of ECM proteins. Renowned for their antioxidant qualities, flavonoids may help to offset the oxidative stress usually linked with bone ailments like osteoporosis. Flavonoids in chitosan scaffolds may help avoid cellular injury by lowering ROS, therefore preserving normal bone regeneration processes. Moreover, the modification of important signaling pathways like MAPK and PI3K/Akt in the presence of chitosan scaffolds loaded with flavonoids emphasizes the significance of these biomaterials in molecularly controlling bone formation and tissue healing [21,31,95,96].

### 3.7. Immunomodulatory Effects

In addition to their antioxidant and anti-inflammatory functions, flavonoid-based biomaterials influence the immune landscape at the injury site—a critical factor in the bone regeneration cascade. While early studies primarily focused on reductions in pro-inflammatory cytokines like TNF-α and IL-6, more recent research highlights the importance of immune cell reprogramming, especially macrophage polarization and its interplay with osteoclasts [97,98].

Macrophages exhibit plasticity between pro-inflammatory M1 and anti-inflammatory M2 phenotypes. M1 macrophages dominate the early inflammatory phase, releasing cytokines such as TNF-α, IL-1β, and IL-6, which can inhibit osteoblast differentiation and promote osteoclastogenesis via RANKL upregulation. Conversely, M2 macrophages secrete IL-10, TGF-β, and VEGF, fostering angiogenesis and osteogenic differentiation. Notably, flavonoids such as quercetin, baicalein, and luteolin have been shown to enhance M2 polarization markers (CD163, CD206) and downregulate M1-associated pathways (e.g., NF-κB signaling) [88,99,100,101].

This immune modulation is tightly coupled with osteoclast behavior, as osteoclasts derive from monocyte/macrophage lineages and are activated in response to pro-inflammatory cues. Thus, biomaterials that favor M2 polarization inherently suppress excessive osteoclastic bone resorption while promoting new bone matrix deposition. Experimental models using flavonoid-functionalized scaffolds have reported reduced TRAP^+^ osteoclast numbers, increased ALP activity, and upregulation of osteogenic genes (RUNX2, OCN) alongside shifts in macrophage phenotype. These findings emphasize that the design of future regenerative platforms must consider immune modulation as a central component rather than a peripheral effect [40,42,102,103].

## 4. Flavonoid Synergism Using MOFs

### 4.1. Flavonoid Coatings in Implantology

One of the major difficulties in bone implantology is achieving fast and consistent osseointegration. Flavonoids—polyphenolic chemicals with varied bioactivities—have been investigated recently as potential biofunctional agents for bioactivate implant surfaces. Particularly in challenged conditions like osteoporotic bone, their antioxidant, anti-inflammatory, osteogenic, and anti-resorptive characteristics make them perfect candidates for coating materials [104,105,106,107,108].

With ALP activity rising by almost 40% relative to control uncoated surfaces, Córdoba et al. (2015) [109] showed that flavonoid-modified titanium surfaces greatly boosted human osteoblast differentiation. Moreover, the over 60% reduction in the release of pro-inflammatory cytokines including IL-6 and TNF-α points to considerable immunomodulating action. Its translational relevance is limited, nonetheless, by the fact that this study focused on in vitro testing and lacked in vivo bone–implant integration data.

Building on particular flavonoid treatments, Llopis-Grimalt et al. (2020) [105] coated porous Ti-6Al-4V implants with quercitrin. Relative to non-coated controls, their in vitro data revealed a 35% increase in osteoblast viability and a 45% decrease in intracellular ROS levels. On surfaces coated with quercitrin, the bacterial adhesion—determined by colony-forming units—dropped by more than half. Notwithstanding these encouraging findings, the study did not evaluate the mechanical stability of the coatings under dynamic stress, an important consideration for clinical use. Loading rutin onto titanium surfaces using a layer-by- layer assembly process, Wu et al. (2024) [110] examined the construction in osteoporotic rat models. After eight weeks, micro-computed tomography (micro-CT) data showed a 32% increase in the bone-volume-to-total-volume ratio (BV/TV) surrounding the coated implants compared to uncoated controls. Histological studies indicated greater BIC, raised by over 28%. Although these findings confirm rutin’s osteopromotive capability, the long-term chemical stability of the layer-by-layer construction is still unknown.

With a 50% decrease in the number of active osteoclasts in vitro and a 45% reduction in peri-implant bone resorptive activity in rat tibiae over a 12-week implantation period, Córdoba et al. (2018) [111] investigated quercitrin nanocoatings and noted a significant reduction in osteoclast resorptive activity. These results are important as, especially in osteoporotic bone, the lifetime of implants depends on the prevention of overly strong osteoclastic activity. But the lack of information from longer implantation times or bigger animal models limits the therapeutic significance.

In an osteoporotic rat model, Wang et al. (2022) [112] explored micro–nano-structured implants loaded with kaempferol. Along with a 20% rise in the BV/TV, their research revealed a 27% increase in maximum pull-out force relative to bare implants. Histological analyses also demonstrated much more ordered bone matrix formation surrounding implants coated with kaempferol. Although the mechanical results are interesting, the research did not investigate the molecular pathways of kaempferol linked with osteogenic signaling completely.

Comparatively, Wang et al. (2021) [113] developed polyelectrolyte multilayers loaded with quercetin and showed, through in vitro assays, that ALP activity was raised by 48% and the expression of Runx2 and OCN was upregulated 2.3-fold and 1.9-fold, respectively, compared to uncoated surfaces under simulated osteoporotic conditions. These findings imply that quercetin maintains osteogenic activity even in a damaged bone environment; long-term in vivo confirmation was not carried out.

Using a different approach, Zhu et al. (2021) [114] coupled strontium and icariin inside TiO_2_ nanotube arrays to produce a 35% higher BV/TV and a 30% increase in mechanical push-out strength compared to non-coated controls in osteoporotic rats. In particular, the dual-agent method yielded better results than any drug by itself, suggesting possible synergistic therapeutic possibilities. Still, the difficulty of dual loading and managing release kinetics might provide major production and regulatory difficulties.

On 3D-printed Ti6Al4V implants, Liu et al. (2022) [115] incorporated quercetin into nano-topographic surfaces. Compared to uncoated nano-topographic implants, their research found a 40% increase in new bone volume and a 35% increase in mechanical fixing strength. Furthermore, about 50% of inflammatory cytokine levels (IL-1β, TNF-α) were lowered by the coating, therefore stressing its dual osteogenic and anti-inflammatory action. Although the combination of surface topography and bioactive compounds seems rather promising, manufacturing expense and complexity might impede practical applicability. Yang et al. (2019) [116] combined polyphenol and gelatin films for implant coatings and showed that ALP activity dropped by 30% in vitro while inflammatory cytokines dropped by up to 40% when compared to gelatin-only coatings. In vivo rat investigations validated these results with notably higher BIC ratios—about a 25% increase. However, the mechanical robustness of the gelatin–polyphenol films under long-term physiological pressure remains unknown.

Finally, some studies—including those by Torre et al. (2018) [117], Weber (2021) [118], and Bjelič and Finšgar (2022) [119]—stress the requirement of multifunctional coatings combining osteopromotive and anti-resorptive characteristics. They do, however, highlight overall that most encouraging findings are still at the preclinical stage and that there is very little movement into human clinical trials (Table 2).

### 4.2. MOFs and Injectable Gels

A modern type of material under investigation for its possibilities in controlled drug delivery and tissue engineering is MOFs. Ideal for continuous drug release and localized treatment uses, MOFs offer a large surface area, adjustable porosity, and amazing biocompatibility. In bone regeneration, where the regulated release of bioactive chemicals may enhance osteogenesis over long periods, these features are particularly important [121,122,123,124].

**Figure 2:**
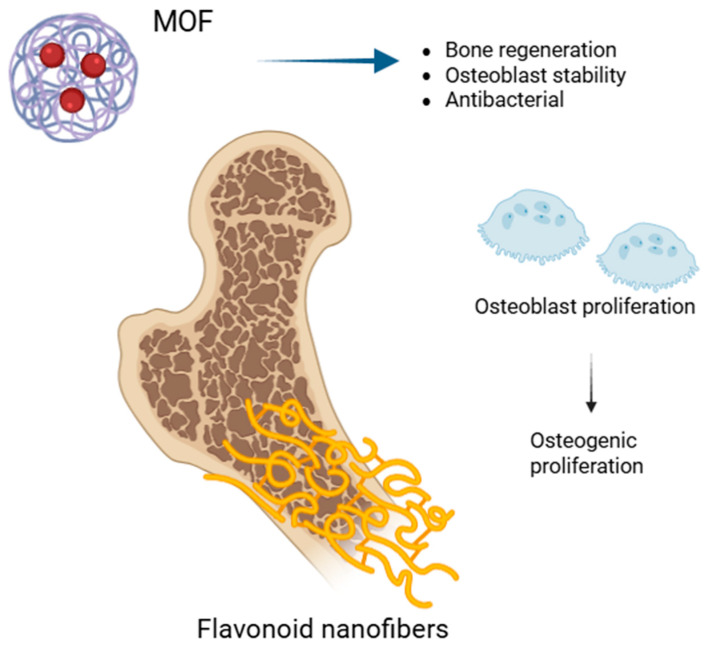
The activity of flavonoid–metal combinations and nanofibers in osteogenesis.

Developing MOF–hydrogel systems—which mix the benefits of MOFs with the bioactive potential of ethnobotanical flavonoids—is one creative approach. Yang et al. (2024) [125] effectively created an MOF–hydrogel system comprising numerous ethnobotanical bioactives, including flavonoids; significant osteogenic, pro-angiogenic, and immunomodulating effects were shown by this system. Treating in vivo rat models with this MOF–flavonoid combination revealed a 72% increase in new bone growth. A key component in obtaining these amazing regeneration results is the MOF’s capacity to provide steady and prolonged release of these bioactives (Figure 2).

Li et al. (2023) [126] created a baicalin-loaded MOF gel system intended to locally block sclerostin (SOST), a negative regulator of bone development, in a similar manner. It has been shown that SOST inhibition improves osteogenesis and accelerates bone formation. In their investigation, rats with calvarial abnormalities had full healing in eight weeks using a baicalin-loaded MOF gel system. These results show the possibility of using MOFs in conjunction with flavonoids to induce bone regeneration, especially in demanding contexts where localized bioactives are essential.

Integrating MOFs with flavonoids allows researchers to create effective approaches for focused, continuous drug release, therefore providing possible answers to the problems of bone regeneration. These mechanisms not only improve osteogenesis but also change the immune response, lower inflammation, and encourage angiogenesis, therefore fostering a more favorable environment for bone repair.

## 5. Conclusions

Despite encouraging preclinical results, the clinical translation of flavonoid-based biomaterials faces several unresolved challenges. These include poor water solubility (typically <10 µg/mL for quercetin, <100 µg/mL for baicalein), chemical instability under physiological pH, and extensive first-pass metabolism, all contributing to extremely low oral bioavailability—often below 2% for flavonoids like quercetin and myricetin. In addition, flavonoids are rapidly cleared from systemic circulation, and their short half-life limits sustained therapeutic efficacy, especially in the context of bone regeneration where long-term signaling is essential.

To overcome these barriers, recent advances have focused on engineering delivery systems that stabilize flavonoids and regulate their release. Metal–flavonoid complexes (e.g., quercetin–Zn^2+^ or baicalein–Ca^2+^) exploit chelation interactions to enhance both antioxidant activity and stability while slowing degradation. Cocrystal formation with pharmaceutically acceptable co-formers such as caffeine or nicotinamide has increased flavonoid solubility 2–4-fold and improved pharmacokinetic parameters (e.g., ~4-fold increase in AUC for baicalein–caffeine). Similarly, phospholipid complexes and polymeric micelles (e.g., Soluplus-based carriers) have increased dissolution rates from <50% to >85% within 30–60 min.

Nonetheless, scalable production remains difficult due to the complexity of encapsulation and the sensitivity of flavonoids to processing conditions like heat, pH, and UV exposure. Regulatory standardization for natural product-based composites is still lacking, and batch-to-batch variability in flavonoid purity may affect consistency in therapeutic outcomes. Addressing these issues requires the establishment of reproducible, GMP-compliant protocols and deeper investigation into flavonoid–matrix binding thermodynamics to design more robust, clinically viable systems.

With an increase of up to 32% in the BV/TV and 28% greater BIC, flavonoid coatings on titanium implants enhanced osteointegration in implantology. Displaying successful osteogenesis and immunomodulation, the addition of flavonoids into MOFs and injectable hydrogels demonstrated a 72% increase in new bone formation in vivo. Though further research is required to confirm long-term clinical effectiveness, these findings show the great promise of flavonoid-functionalized biomaterials in bone regeneration.

## Figures and Tables

**Table 1:** Flavonoid–metal complexes and their osteogenic effects.

Flavonoid	Metal Ion	System Type	Key Findings	Reference
Quercetin	Cu(II)	Hydrogel/Complex	↑ VEGF, ↑ ALP (45%), ↑ OCN (osteogenesis)	[48]
Kaempferol	Zn(II)	Zebrafish Model	↑ Bone mineralization, enhanced vertebral regeneration	[69]
Morin	Zn(II)	In Vitro Culture	↑ ALP, ↑ COL1A1, enhanced osteogenic differentiation	[70]
Rutin	Zn(II)	hDPSC Culture	↑ Mineralization, osteogenic gene expression	[71]
Silibinin	Cu(II)	In Vitro Culture	↑ Osteogenesis (1.6×), increased OCN, ALP expression	[72]

↑—increase.

**Table 2:** Summary of flavonoid-functionalized surfaces of implants.

Flavonoid/Agent	Experimental Models	Main Quantitative Outcomes	Limitations/Comments	Reference
Flavonoid derivatives	In vitro (osteoblasts, fibroblasts)	+40% ALP activity; −60% IL-6/TNF-α secretion	No in vivo data; only short-term assays	[120]
Quercitrin	In vitro (osteoblasts)	+35% osteoblast viability; −50% bacterial adhesion	Lacked mechanical evaluation	[105]
Rutin	In vivo (osteoporotic rats)	+32% BV/TV; +28% BIC	Layer stability over time uncertain	[110]
Quercitrin	In vitro and in vivo (rat tibia)	−50% osteoclast activity; −45% bone resorption	No large animal models	[111]
Kaempferol	In vivo (osteoporotic rats)	+27% pull-out force; +20% BV/TV	Lack of mechanistic pathway elucidation	[112]
Quercetin	In vitro (osteoporotic-like conditions)	+48% ALP activity; 2.3-fold Runx2 increase	No in vivo confirmation	[113]
Strontium + icariin	In vitro and in vivo (osteoporotic rats)	+35% BV/TV; +30% push-out strength	Manufacturing complexity	[114]
Quercetin	In vitro and in vivo	+40% bone volume; −50% IL-1β, TNF-α levels	High cost and technical complexity	[115]
Polyphenol + gelatin	In vitro and in vivo	+30% ALP; +25% BIC	Mechanical resilience questionable	[116]

## Abbreviations

The following abbreviations are used in this manuscript

ALP	Alkaline Phosphatase
BIC	Bone–Implant Contact
BV/TV	Bone-Volume-to-Total-Volume Ratio
ECM	Extracellular Matrix
EGCG	Epigallocatechin Gallate
HA	Hydroxyapatite
hMSC	Human Mesenchymal Stem Cell
MOFs	Metal–Organic Frameworks
OCN	Osteocalcin
OPN	Osteopontin
PCL	Polycaprolactone
ROS	Reactive Oxygen Species
RUNX2	Runt-Related Transcription Factor 2
TNF-α	Tumor Necrosis Factor Alpha

## References

[1] Shen Y., Huang X., Wu J., Lin X., Zhou X., Zhu Z., Pan X., Xu J., Qiao J., Zhang T. (2022). The Global Burden of Osteoporosis, Low Bone Mass, and Its Related Fracture in 204 Countries and Territories, 1990–2019. Front. Endocrinol..

[2] Zhu Z., Yu P., Wu Y., Wu Y., Tan Z., Ling J., Ma J., Zhang J., Zhu W., Liu X. (2023). Sex Specific Global Burden of Osteoporosis in 204 Countries and Territories, from 1990 to 2030: An Age-Period-Cohort Modeling Study. J. Nutr. Health Aging.

[3] Lorentzon M., Johansson H., Harvey N.C., Liu E., Vandenput L., McCloskey E.V., Kanis J.A. (2022). Osteoporosis and Fractures in Women: The Burden of Disease. Climacteric.

[4] Feng J., Zhang C., Li B., Zhan S., Wang S., Song C. (2024). Global Burden of Hip Fracture: The Global Burden of Disease Study. Osteoporos. Int..

[5] Wu A.-M., Bisignano C., James S.L., Abady G.G., Abedi A., Abu-Gharbieh E., Alhassan R.K., Alipour V., Arabloo J., Asaad M. (2021). Global, Regional, and National Burden of Bone Fractures in 204 Countries and Territories, 1990–2019: A Systematic Analysis from the Global Burden of Disease Study 2019. Lancet Healthy Longev..

[6] Salari N., Ghasemi H., Mohammadi L., Behzadi M.H., Rabieenia E., Shohaimi S., Mohammadi M. (2021). The Global Prevalence of Osteoporosis in the World: A Comprehensive Systematic Review and Meta-Analysis. J. Orthop. Surg. Res..

[7] Rashidi M.-M., Saeedi Moghaddam S., Azadnajafabad S., Heidari-Foroozan M., Hashemi S.M., Mohammadi E., Esfahani Z., Ebrahimi N., Shobeiri P., Malekpour M.-R. (2023). Low Bone Mineral Density, a Neglected Condition in North Africa and Middle East: Estimates from the Global Burden of Disease Study, 1990–2019. Osteoporos. Int..

[8] Curtis E.M., Harvey N.C., Cooper C. (2018). The Burden of Osteoporosis. Osteoporosis.

[9] Dong Y., Peng R., Kang H., Song K., Guo Q., Zhao H., Zhu M., Zhang Y., Guan H., Li F. (2022). Global Incidence, Prevalence, and Disability of Vertebral Fractures: A Systematic Analysis of the Global Burden of Disease Study 2019. Spine J..

[10] Xiao P.-L., Cui A.-Y., Hsu C.-J., Peng R., Jiang N., Xu X.-H., Ma Y.-G., Liu D., Lu H.-D. (2022). Global, Regional Prevalence, and Risk Factors of Osteoporosis According to the World Health Organization Diagnostic Criteria: A Systematic Review and Meta-Analysis. Osteoporos. Int..

[11] Marcellusi A., Rotundo M.A., Nardone C., Sciattella P., Gazzillo S., Rossini M., Barbagallo M., Antenori A., Valle D., Mennini F.S. (2020). Osteoporosis: Economic Burden of Disease in Italy. Clin. Drug Investig..

[12] Agrawal A.C., Garg A.K. (2023). Epidemiology of Osteoporosis. Indian J. Orthop..

[13] Rozenberg S., Bruyère O., Bergmann P., Cavalier E., Gielen E., Goemaere S., Kaufman J.M., Lapauw B., Laurent M.R., De Schepper J. (2020). How to Manage Osteoporosis before the Age of 50. Maturitas.

[14] Ebeling P.R., Nguyen H.H., Aleksova J., Vincent A.J., Wong P., Milat F. (2022). Secondary Osteoporosis. Endocr. Rev..

[15] Foessl I., Dimai H.P., Obermayer-Pietsch B. (2023). Long-Term and Sequential Treatment for Osteoporosis. Nat. Rev. Endocrinol..

[16] Gao Y., Patil S., Jia J. (2021). The Development of Molecular Biology of Osteoporosis. Int. J. Mol. Sci..

[17] Lungu I.I., Stefanache A., Crivoi F., BUREC A.-F., Belei D., Cioanca O., Hancianu M. (2024). Innovative Synthesis of Zinc and Selenium Complexes with Gallic Acid: Exploring Their Antioxidant Potential. Med.-Surg. J..

[18] Andia I., Maffulli N. (2013). Platelet-Rich Plasma for Managing Pain and Inflammation in Osteoarthritis. Nat. Rev. Rheumatol..

[19] De Pace R., Molinari S., Mazzoni E., Perale G. (2025). Bone Regeneration: A Review of Current Treatment Strategies. J. Clin. Med..

[20] Laubach M., Hildebrand F., Suresh S., Wagels M., Kobbe P., Gilbert F., Kneser U., Holzapfel B.M., Hutmacher D.W. (2023). The Concept of Scaffold-Guided Bone Regeneration for the Treatment of Long Bone Defects: Current Clinical Application and Future Perspective. J. Funct. Biomater..

[21] Yang J., Zhang L., Ding Q., Zhang S., Sun S., Liu W., Liu J., Han X., Ding C. (2023). Flavonoid-Loaded Biomaterials in Bone Defect Repair. Molecules.

[22] Mei H., Liu H., Sha C., Lv Q., Song Q., Jiang L., Tian E., Gao Z., Li J., Zhou J. (2024). Multifunctional Metal–Phenolic Composites Promote Efficient Periodontitis Treatment via Antibacterial and Osteogenic Properties. ACS Appl. Mater. Interfaces.

[23] Makvandi P., Ghomi M., Padil V.V.T., Shalchy F., Ashrafizadeh M., Askarinejad S., Pourreza N., Zarrabi A., Nazarzadeh Zare E., Kooti M. (2020). Biofabricated Nanostructures and Their Composites in Regenerative Medicine. ACS Appl. Nano Mater..

[24] Shen N., Wang T., Gan Q., Liu S., Wang L., Jin B. (2022). Plant Flavonoids: Classification, Distribution, Biosynthesis, and Antioxidant Activity. Food Chem..

[25] Roy A., Khan A., Ahmad I., Alghamdi S., Rajab B.S., Babalghith A.O., Alshahrani M.Y., Islam S., Islam M.R. (2022). Flavonoids a Bioactive Compound from Medicinal Plants and Its Therapeutic Applications. BioMed Res. Int..

[26] Dias M.C., Pinto D.C.G.A., Silva A.M.S. (2021). Plant Flavonoids: Chemical Characteristics and Biological Activity. Molecules.

[27] Liga S., Paul C., Péter F. (2023). Flavonoids: Overview of Biosynthesis, Biological Activity, and Current Extraction Techniques. Plants.

[28] Teng H., Zheng Y., Cao H., Huang Q., Xiao J., Chen L. (2023). Enhancement of Bioavailability and Bioactivity of Diet-Derived Flavonoids by Application of Nanotechnology: A Review. Crit. Rev. Food Sci. Nutr..

[29] Wang L., Song J., Liu A., Xiao B., Li S., Wen Z., Lu Y., Du G. (2020). Research Progress of the Antiviral Bioactivities of Natural Flavonoids. Nat. Prod. Bioprospect..

[30] Rédai E.-M., Antonoaea P., Todoran N., Vlad R.A., Bîrsan M., Tătaru A., Ciurba A. (2021). Development and Evaluation of Fluoxetine Fast Dissolving Films: An Alternative for Noncompliance in Pediatric Patients. Processes.

[31] Shanmugavadivu A., Balagangadharan K., Selvamurugan N. (2022). Angiogenic and Osteogenic Effects of Flavonoids in Bone Regeneration. Biotechnol. Bioeng..

[32] Zhang J., Liu Z., Luo Y., Li X., Huang G., Chen H., Li A., Qin S. (2022). The Role of Flavonoids in the Osteogenic Differentiation of Mesenchymal Stem Cells. Front. Pharmacol..

[33] Li J., Wang X., Wang Y., Lu C., Zheng D., Zhang J. (2019). Isoquercitrin, a Flavonoid Glucoside, Exerts a Positive Effect on Osteogenesis In Vitro and In Vivo. Chem.-Biol. Interact..

[34] Sharma A.R., Nam J.-S. (2019). Kaempferol Stimulates WNT/β-Catenin Signaling Pathway to Induce Differentiation of Osteoblasts. J. Nutr. Biochem..

[35] Guo A.J.Y., Choi R.C.Y., Cheung A.W.H., Chen V.P., Xu S.L., Dong T.T.X., Chen J.J., Tsim K.W.K. (2011). Baicalin, a Flavone, Induces the Differentiation of Cultured Osteoblasts: An Action via the Wnt/β-CATENIN Signaling Pathway * ♦. J. Biol. Chem..

[36] Li S., Zhou H., Hu C., Yang J., Ye J., Zhou Y., Li Z., Chen L., Zhou Q. (2021). Total Flavonoids of *Rhizoma drynariae* Promotes Differentiation of Osteoblasts and Growth of Bone Graft in Induced Membrane Partly by Activating Wnt/β-Catenin Signaling Pathway. Front. Pharmacol..

[37] Sharma A.R., Lee Y.-H., Bat-Ulzii A., Chatterjee S., Bhattacharya M., Chakraborty C., Lee S.-S. (2023). Bioactivity, Molecular Mechanism, and Targeted Delivery of Flavonoids for Bone Loss. Nutrients.

[38] Li Z. (2024). Investigation of the Molecular Mechanism of Quercetin in Inhibiting Ankylosing Spondylitis Ossification via the Bone Morphogenetic Protein/Smad Signaling Pathway. Med. Mol. Morphol..

[39] Sheibani M., Shayan M., Jafari-Sabet M., Sharifi A.M. (2023). Applications of Phytomedicines in Chondrocytes and Osteocytes Regeneration Therapy: Pre-Clinical and Clinical Studies. Tradit. Integr. Med..

[40] Su H., Liu L., Yan Z., Guo W., Huang G., Zhuang R., Pan Y. (2025). Therapeutic Potential of Total Flavonoids of *Rhizoma drynariae*: Inhibiting Adipogenesis and Promoting Osteogenesis via MAPK/HIF-1α Pathway in Primary Osteoporosis. J. Orthop. Surg. Res..

[41] Zha X., Xu Z., Liu Y., Xu L., Huang H., Zhang J., Cui L., Zhou C., Xu D. (2016). Amentoflavone Enhances Osteogenesis of Human Mesenchymal Stem Cells through JNK and P38 MAPK Pathways. J. Nat. Med..

[42] Ramesh P., Jagadeesan R., Sekaran S., Dhanasekaran A., Vimalraj S. (2021). Flavonoids: Classification, Function, and Molecular Mechanisms Involved in Bone Remodelling. Front. Endocrinol..

[43] Tian W., Zhang W., Chen L., Liao J., Yang X. (2024). Bioflavonoid Tangeretin Regulates RANK/RANKL/OPG Signaling Proteins via Stimulating Estrogenic Activity in Ovariectomized Rats. Pharmacogn. Mag..

[44] Bellavia D., Dimarco E., Costa V., Carina V., Luca A.D., Raimondi L., Fini M., Gentile C., Caradonna F., Giavaresi G. (2021). Flavonoids in Bone Erosive Diseases: Perspectives in Osteoporosis Treatment. Trends Endocrinol. Metab..

[45] Jin H., Jiang N., Xu W., Zhang Z., Yang Y., Zhang J., Xu H. (2022). Effect of Flavonoids from *Rhizoma drynariae* on Osteoporosis Rats and Osteocytes. Biomed. Pharmacother..

[46] Rodríguez V., Rivoira M., Picotto G., de Barboza G.D., Collin A., Tolosa de Talamoni N. (2022). Analysis of the Molecular Mechanisms by Flavonoids with Potential Use for Osteoporosis Prevention or Therapy. Curr. Med. Chem..

[47] Du J., Wang Y., Wu C., Zhang X., Zhang X., Xu X. (2024). Targeting Bone Homeostasis Regulation: Potential of Traditional Chinese Medicine Flavonoids in the Treatment of Osteoporosis. Front. Pharmacol..

[48] Vimalraj S., Rajalakshmi S., Raj Preeth D., Vinoth Kumar S., Deepak T., Gopinath V., Murugan K., Chatterjee S. (2018). Mixed-Ligand Copper(II) Complex of Quercetin Regulate Osteogenesis and Angiogenesis. Mater. Sci. Eng. C.

[49] Kejík Z., Kaplánek R., Masařík M., Babula P., Matkowski A., Filipenský P., Veselá K., Gburek J., Sýkora D., Martásek P. (2021). Iron Complexes of Flavonoids-Antioxidant Capacity and Beyond. Int. J. Mol. Sci..

[50] Al-Garawi Z.S., Al-Qaisi A.H.I., Al-Shamari K.A., Öztürkkan F.E., Necefoğlu H. (2024). The Utility of *Hibiscus sabdariffa* L. to Prepare Metal Oxides NPs for Clinical Application on Osteoporosis Supported by Theoretical Study. Bioprocess. Biosyst. Eng..

[51] Gaddi G.M., Caro-Ramírez J.Y., Parente J.E., Williams P.A.M., Ferrer E.G. (2023). Copper-Flavonoid Family of Complexes Involved in Alkaline Phosphatase Activation. Biometals.

[52] Walencik P.K., Choińska R., Gołębiewska E., Kalinowska M. (2024). Metal–Flavonoid Interactions—From Simple Complexes to Advanced Systems. Molecules.

[53] Khater M., Ravishankar D., Greco F., Osborn H.M. (2019). Metal Complexes of Flavonoids: Their Synthesis, Characterization and Enhanced Antioxidant and Anticancer Activities. Future Med. Chem..

[54] Rodríguez-Arce E., Saldías M. (2021). Antioxidant Properties of Flavonoid Metal Complexes and Their Potential Inclusion in the Development of Novel Strategies for the Treatment against Neurodegenerative Diseases. Biomed. Pharmacother..

[55] Sun H., Chen Y., Sang X., Liu Q., Yu H., Hu S., Mao Y., Zhang L. (2025). Spatiotemporal Regulation of the Bone Immune Microenvironment via a ‘Zn^2+^-Quercetin’ Hierarchical Delivery System for Bone Regeneration. Regen. Biomater..

[56] Raj Preeth D., Saravanan S., Shairam M., Selvakumar N., Selestin Raja I., Dhanasekaran A., Vimalraj S., Rajalakshmi S. (2021). Bioactive Zinc(II) Complex Incorporated PCL/Gelatin Electrospun Nanofiber Enhanced Bone Tissue Regeneration. Eur. J. Pharm. Sci..

[57] Xue D., Chen E., Zhang W., Gao X., Wang S., Zheng Q., Pan Z., Li H., Liu L. (2017). The Role of Hesperetin on Osteogenesis of Human Mesenchymal Stem Cells and Its Function in Bone Regeneration. Oncotarget.

[58] Mancim-Imbriani M.J., Duarte J.L., Di Filippo L.D., Durão L.P.L., Chorilli M., Palomari Spolidorio D.M., Maquera-Huacho P.M. (2024). Formulation of a Novel Hesperetin-Loaded Nanoemulsion and Its Promising Effect on Osteogenesis. Pharmaceutics.

[59] Ortiz A.D.C., Fideles S.O.M., Reis C.H.B., Bellini M.Z., Pereira E.d.S.B.M., Pilon J.P.G., de Marchi M.Â., Detregiachi C.R.P., Flato U.A.P., Trazzi B.F.d.M. (2022). Therapeutic Effects of Citrus Flavonoids Neohesperidin, Hesperidin and Its Aglycone, Hesperetin on Bone Health. Biomolecules.

[60] Li W., Pi J., Zhang Y., Ma X., Zhang B., Wang S., Qi D., Li N., Guo P., Liu Z. (2018). A Strategy to Improve the Oral Availability of Baicalein: The Baicalein-Theophylline Cocrystal. Fitoterapia.

[61] Guan D., Xuan B., Wang C., Long R., Jiang Y., Mao L., Kang J., Wang Z., Chow S.F., Zhou Q. (2021). Improving the Physicochemical and Biopharmaceutical Properties of Active Pharmaceutical Ingredients Derived from Traditional Chinese Medicine through Cocrystal Engineering. Pharmaceutics.

[62] You G., Feng T., Zhang G., Chen M., Liu F., Sun L., Wang M., Ren X. (2021). Preparation, Optimization, Characterization and In Vitro Release of Baicalein-Solubilizing Glycyrrhizic Acid Nano-Micelles. Int. J. Pharm..

[63] Fan J., Dai Y., Shen H., Ju J., Zhao Z. (2017). Application of Soluplus to Improve the Flowability and Dissolution of Baicalein Phospholipid Complex. Molecules.

[64] Zhang B., Tang L., Tian F., Ding Q., Hu Z., Wang J.-R., Mei X. (2024). Rutin Cocrystals with Improved Solubility, Bioavailability, and Bioactivities. Cryst. Growth Des..

[65] Smith A.J., Kavuru P., Wojtas L., Zaworotko M.J., Shytle R.D. (2011). Cocrystals of Quercetin with Improved Solubility and Oral Bioavailability. Mol. Pharm..

[66] Wu Y.-W., Chen S.-C., Lai W.-F.T., Chen Y.-C., Tsai Y.-H. (2013). Screening of Flavonoids for Effective Osteoclastogenesis Suppression. Anal. Biochem..

[67] Huh J.-E., Jung I.-T., Choi J., Baek Y.-H., Lee J.-D., Park D.-S., Choi D.-Y. (2013). The Natural Flavonoid Galangin Inhibits Osteoclastic Bone Destruction and Osteoclastogenesis by Suppressing NF-κB in Collagen-Induced Arthritis and Bone Marrow-Derived Macrophages. Eur. J. Pharmacol..

[68] Zhang J.-F., Li G., Meng C.-L., Dong Q., Chan C.-Y., He M.-L., Leung P.-C., Zhang Y.-O., Kung H.-F. (2009). Total Flavonoids of *Herba Epimedii* Improves Osteogenesis and Inhibits Osteoclastogenesis of Human Mesenchymal Stem Cells. Phytomedicine.

[69] Vimalraj S., Saravanan S., Hariprabu G., Yuvashree R., Ajieth Kanna S.K., Sujoy K., Anjali D. (2020). Kaempferol-Zinc(II) Complex Synthesis and Evaluation of Bone Formation Using Zebrafish Model. Life Sci..

[70] Vimalraj S., Rajalakshmi S., Saravanan S., Thirumalai D., Kadarkarai M., Rajkumar A.V., Dhanasekaran A. (2019). Zinc Chelated Morin Promotes Osteoblast Differentiation over Its Uncomplexed Counterpart. Process Biochem..

[71] Vimalraj S., Saravanan S., Subramanian R. (2021). Rutin-Zn(II) Complex Promotes Bone Formation—A Concise Assessment in Human Dental Pulp Stem Cells and Zebrafish. Chem.-Biol. Interact..

[72] Rajalakshmi S., Vimalraj S., Saravanan S., Raj Preeth D., Shairam M., Anuradha D. (2018). Synthesis and Characterization of Silibinin/Phenanthroline/Neocuproine Copper(II) Complexes for Augmenting Bone Tissue Regeneration: An In Vitro Analysis. J. Biol. Inorg. Chem..

[73] Han C., Guo M., Bai J., Zhao L., Wang L., Song W., Zhang P. (2022). Quercetin-Loaded Nanocomposite Microspheres for Chronologically Promoting Bone Repair via Synergistic Immunoregulation and Osteogenesis. Mater. Des..

[74] Yang J., Zhang L., Wang Y., Wang N., Wei H., Zhang S., Ding Q., Sun S., Ding C., Liu W. (2024). Dihydromyricetin-Loaded Oxidized Polysaccharide/L-Arginine Chitosan Adhesive Hydrogel Promotes Bone Regeneration by Regulating PI3K/AKT Signaling Pathway and MAPK Signaling Pathway. Carbohydr. Polym..

[75] Li D., Yin W., Xu C., Feng Y., Huang X., Hao J., Zhu C. (2024). Rutin Promotes Osteogenic Differentiation of Mesenchymal Stem Cells (MSCs) by Increasing ECM Deposition and Inhibiting P53 Expression. Aging.

[76] Selvaraj V., Subramanian R., Sekaran S., Veeraiyan D.N., Thangavelu L. (2021). Ferulic Acid-Cu(II) and Zn(II) Complexes Promote Bone Formation. Process Biochem..

[77] Fernández-Villa D., Aguilar M.R., Rojo L. (2024). Europium–Tannic Acid Nanocomplexes Devised for Bone Regeneration under Oxidative or Inflammatory Environments. J. Mater. Chem. B.

[78] Kim H., Lee H., Knowles J.C. (2006). Electrospinning Biomedical Nanocomposite Fibers of Hydroxyapatite/Poly(Lactic Acid) for Bone Regeneration. J. Biomed. Mater. Res..

[79] Shetty K., Bhandari A., Yadav K.S. (2022). Nanoparticles Incorporated in Nanofibers Using Electrospinning: A Novel Nano-in-Nano Delivery System. J. Control. Release.

[80] Shin S.-H., Purevdorj O., Castano O., Planell J.A., Kim H.-W. (2012). A Short Review: Recent Advances in Electrospinning for Bone Tissue Regeneration. J. Tissue Eng..

[81] Wang Z., Wang Y., Yan J., Zhang K., Lin F., Xiang L., Deng L., Guan Z., Cui W., Zhang H. (2021). Pharmaceutical Electrospinning and 3D Printing Scaffold Design for Bone Regeneration. Adv. Drug Deliv. Rev..

[82] Raja I.S., Preeth D.R., Vedhanayagam M., Hyon S.-H., Lim D., Kim B., Rajalakshmi S., Han D.-W. (2021). Polyphenols-Loaded Electrospun Nanofibers in Bone Tissue Engineering and Regeneration. Biomater. Res..

[83] Hoveidaei A.H., Sadat-Shojai M., Mosalamiaghili S., Salarikia S.R., Roghani-shahraki H., Ghaderpanah R., Ersi M.H., Conway J.D. (2024). Nano-Hydroxyapatite Structures for Bone Regenerative Medicine: Cell-Material Interaction. Bone.

[84] Bal Z., Kaito T., Korkusuz F., Yoshikawa H. (2020). Bone Regeneration with Hydroxyapatite-Based Biomaterials. Emergent Mater..

[85] Abere D.V., Ojo S.A., Oyatogun G.M., Paredes-Epinosa M.B., Niluxsshun M.C.D., Hakami A. (2022). Mechanical and Morphological Characterization of Nano-Hydroxyapatite (nHA) for Bone Regeneration: A Mini Review. Biomed. Eng. Adv..

[86] Florea D.A., Chircov C., Grumezescu A.M. (2020). Hydroxyapatite Particles—Directing the Cellular Activity in Bone Regeneration Processes: An Up-To-Date Review. Appl. Sci..

[87] Costache A.-D., Leon-Constantin M.-M., Roca M., Maștaleru A., Anghel R.-C., Zota I.-M., Drugescu A., Costache I.-I., Chetran A., Moisă Ș.-M. (2022). Cardiac Biomarkers in Sports Cardiology. J. Cardiovasc. Dev. Dis..

[88] Forte L., Torricelli P., Boanini E., Gazzano M., Rubini K., Fini M., Bigi A. (2016). Antioxidant and Bone Repair Properties of Quercetin-Functionalized Hydroxyapatite: An In Vitro Osteoblast–Osteoclast–Endothelial Cell Co-Culture Study. Acta Biomater..

[89] Tao F., Cheng Y., Shi X., Zheng H., Du Y., Xiang W., Deng H. (2020). Applications of Chitin and Chitosan Nanofibers in Bone Regenerative Engineering. Carbohydr. Polym..

[90] Kudiyarasu S., Karuppan Perumal M.K., Rajan Renuka R., Manickam Natrajan P. (2024). Chitosan Composite with Mesenchymal Stem Cells: Properties, Mechanism, and Its Application in Bone Regeneration. Int. J. Biol. Macromol..

[91] Fasolino I., Raucci M.G., Soriente A., Demitri C., Madaghiele M., Sannino A., Ambrosio L. (2019). Osteoinductive and Anti-Inflammatory Properties of Chitosan-Based Scaffolds for Bone Regeneration. Mater. Sci. Eng. C.

[92] Dmour B.-A., Costache A.D., Dmour A., Huzum B., Duca Ș.T., Chetran A., Miftode R.Ș., Afrăsânie I., Tuchiluș C., Cianga C.M. (2023). Could Endothelin-1 Be a Promising Neurohormonal Biomarker in Acute Heart Failure?. Diagnostics.

[93] Yang Y., Sun W., Fu Q., Wang Z., Zhao H., Wang Z., Gao Y., Wang J. (2024). Fabrication and Evaluation of Zn-EGCG-Loaded Chitosan Scaffolds for Bone Regeneration: From Cellular Responses to in Vivo Performance. Int. J. Biol. Macromol..

[94] Li Y., Selvaraj V., Saravanan S., Abullais S.S., Wankhade V. (2024). Exploring the Osteogenic Potential of Chitosan-Quercetin Bio-Conjugate: In Vitro and In Vivo Investigations in Osteoporosis Models. Int. J. Biol. Macromol..

[95] Valentino A., Di Cristo F., Bosetti M., Amaghnouje A., Bousta D., Conte R., Calarco A. (2021). Bioactivity and Delivery Strategies of Phytochemical Compounds in Bone Tissue Regeneration. Appl. Sci..

[96] Lungu I.I., Cioanca O., Mircea C., Tuchilus C., Stefanache A., Huzum R., Hancianu M. (2024). Insights into Catechin–Copper Complex Structure and Biologic Activity Modulation. Molecules.

[97] Chen S., Zhang H., Wang Z., Zhu D., Li Y., Zhang Y., Wang D., Chen S., Liu H., Kang X. (2025). Macrophage Efferocytosis as a Therapeutic Strategy in Intervertebral Disc Degeneration. Cell Prolif..

[98] Radandish M., Khalilian P., Esmaeil N. (2021). The Role of Distinct Subsets of Macrophages in the Pathogenesis of MS and the Impact of Different Therapeutic Agents on These Populations. Front. Immunol..

[99] Tang M., Wang G., Li J., Wang Y., Peng C., Chang X., Guo J., Gui S. (2024). Flavonoid Extract from Propolis Alleviates Periodontitis by Boosting Periodontium Regeneration and Inflammation Resolution via Regulating TLR4/MyD88/NF-*κ*B and RANK/NF-*κ*B Pathway. J. Ethnopharmacol..

[100] Zhou X., Zhang Z., Jiang W., Hu M., Meng Y., Li W., Zhou X., Wang C. (2022). Naringenin Is a Potential Anabolic Treatment for Bone Loss by Modulating Osteogenesis, Osteoclastogenesis, and Macrophage Polarization. Front. Pharmacol..

[101] Ge Y.-W., Feng K., Liu X.-L., Zhu Z.-A., Chen H.-F., Chang Y.-Y., Sun Z.-Y., Wang H.-W., Zhang J.-W., Yu D.-G. (2020). Quercetin Inhibits Macrophage Polarization through the P-38α/β Signalling Pathway and Regulates OPG/RANKL Balance in a Mouse Skull Model. J. Cell. Mol. Med..

[102] Xu B., Wang X., Wu C., Zhu L., Chen O., Wang X. (2018). Flavonoid Compound Icariin Enhances BMP-2 Induced Differentiation and Signalling by Targeting to Connective Tissue Growth Factor (CTGF) in SAMP6 Osteoblasts. PLoS ONE.

[103] Zhang Y., Wang Q., Pan H., Xu H., Deng W., Sun X. (2024). *Panicum miliaceum* L. and Bone Healing Properties in Rat Model of Femur Fracture by Activating Phosphate Stimulating Macrophages via BMP2 and RANK Signaling Pathway. Pharmacogn. Mag..

[104] Krasnikov A., Krasnikova E., Morozova D., Spirkina N. (2022). Study of Osseointegration Properties of Multilayer Coatings with a Biodegradable Film of Flavonoid Nanoaggregates. J. Phys. Conf. Ser..

[105] Llopis-Grimalt M.A., Arbós A., Gil-Mir M., Mosur A., Kulkarni P., Salito A., Ramis J.M., Monjo M. (2020). Multifunctional Properties of Quercitrin-Coated Porous Ti-6Al-4V Implants for Orthopaedic Applications Assessed In Vitro. J. Clin. Med..

[106] Liu L., Lan X., Chen X., Dai S., Wang Z., Zhao A., Lu L., Huang N., Chen J., Yang P. (2023). Multi-Functional Plant Flavonoids Regulate Pathological Microenvironments for Vascular Stent Surface Engineering. Acta Biomater..

[107] Arias-Mainer C., Romero-Gavilán F., Cerqueira A., Peñarrocha-Oltra D., García-Arnáez I., Amorrortu O., Azkargorta M., Elortza F., Gurruchaga M., Goni I. (2025). Quercetin-Doped Sol-Gel Coatings on Titanium Implants: A Promising Approach for Enhanced Immune Response and Cell Adhesion. J. Mater. Chem. B.

[108] Wang Y., Duan H., Zhang Z., Chen L., Li J. (2024). Research Progress on the Application of Natural Medicines in Biomaterial Coatings. Materials.

[109] Córdoba A., Satué M., Gómez-Florit M., Monjo M., Ramis J.M. (2015). Flavonoid Coated Titanium Surfaces for Bioactive Bone Implants. Stem Cell Transl. Investig..

[110] Wu Y., Wang Y., Chen F., Wang B. (2024). Loading Rutin on Surfaces by the Layer-by-Layer Assembly Technique to Improve the Oxidation Resistance and Osteogenesis of Titanium Implants in Osteoporotic Rats. Biomed. Mater..

[111] Córdoba A., Manzanaro-Moreno N., Colom C., Rønold H.J., Lyngstadaas S.P., Monjo M., Ramis J.M. (2018). Quercitrin Nanocoated Implant Surfaces Reduce Osteoclast Activity In Vitro and In Vivo. Int. J. Mol. Sci..

[112] Wang A., Yuan W., Song Y., Zang Y., Yu Y. (2022). Osseointegration Effect of Micro-Nano Implants Loaded With Kaempferol in Osteoporotic Rats. Front. Bioeng. Biotechnol..

[113] Wang B., Chen L., Xie J., Tang J., Hong C., Fang K., Jin C., Huang C., Xu T., Yang L. (2021). Coating Polyelectrolyte Multilayers Loaded with Quercetin on Titanium Surfaces by Layer-By-Layer Assembly Technique to Improve Surface Osteogenesis Under Osteoporotic Condition. J. Biomed. Nanotechnol..

[114] Zhu Y., Zheng T., Wen L., Li R., Zhang Y., Bi W., Feng X., Qi M. (2021). Osteogenic Capability of Strontium and Icariin-Loaded TiO2 Nanotube Coatings in Vitro and in Osteoporotic Rats. J. Biomater. Appl..

[115] Liu N., Wang H., Fu Z., Zhang C., Hui W., Wu J., Zhang Y., Zhang S. (2022). Quercetin-Coating Promotes Osteogenic Differentiation, Osseointegration and Anti-Inflammatory Properties of Nano-Topographic Modificated 3D-Printed Ti6Al4V Implant. Front. Bioeng. Biotechnol..

[116] Yang S., Wang Y., Luo S., Shan C., Geng Y., Zhang T., Sheng S., Zan X. (2019). Building Polyphenol and Gelatin Films as Implant Coating, Evaluating from in Vitro and in Vivo Performances. Colloids Surf. B Biointerfaces.

[117] Torre E., Iviglia G., Cassinelli C., Morra M. (2018). Potentials of Polyphenols in Bone-Implant Devices.

[118] Weber F. (2021). Development of Multifunctional Polyphenolic Coatings for Improved Peri-Implant Healing. Ph.D. Thesis.

[119] Bjelič D., Finšgar M. (2022). Bioactive Coatings with Anti-Osteoclast Therapeutic Agents for Bone Implants: Enhanced Compliance and Prolonged Implant Life. Pharmacol. Res..

[120] Córdoba A., Satué M., Gómez-Florit M., Hierro-Oliva M., Petzold C., Lyngstadaas S.P., González-Martín M.L., Monjo M., Ramis J.M. (2015). Flavonoid-Modified Surfaces: Multifunctional Bioactive Biomaterials with Osteopromotive, Anti-Inflammatory, and Anti-Fibrotic Potential. Adv. Healthc. Mater..

[121] Xue Y., Zhu Z., Zhang X., Chen J., Yang X., Gao X., Zhang S., Luo F., Wang J., Zhao W. (2021). Accelerated Bone Regeneration by MOF Modified Multifunctional Membranes through Enhancement of Osteogenic and Angiogenic Performance. Adv. Healthc. Mater..

[122] Chen M., Wang D., Li M., He Y., He T., Chen M., Hu Y., Luo Z., Cai K. (2022). Nanocatalytic Biofunctional MOF Coating on Titanium Implants Promotes Osteoporotic Bone Regeneration through Cooperative Pro-Osteoblastogenesis MSC Reprogramming. ACS Nano.

[123] Yu S., Wu T., Xu K., Liu R., Yu T., Wang Z., Zhang Z. (2025). 3D Bioprinted Biomimetic MOF-Functionalized Hydrogel Scaffolds for Bone Regeneration: Synergistic Osteogenesis and Osteoimmunomodulation. Mater. Today Bio.

[124] Si Y., Liu H., Yu H., Jiang X., Sun D. (2022). MOF-Derived CuO@ZnO Modified Titanium Implant for Synergistic Antibacterial Ability, Osteogenesis and Angiogenesis. Colloids Surf. B Biointerfaces.

[125] Yang S., Zhu Y., Ji C., Zhu H., Lao A., Zhao R., Hu Y., Zhou Y., Zhou J., Lin K. (2024). A Five-in-One Novel MOF-Modified Injectable Hydrogel with Thermo-Sensitive and Adhesive Properties for Promoting Alveolar Bone Repair in Periodontitis: Antibacterial, Hemostasis, Immune Reprogramming, pro-Osteo-/Angiogenesis and Recruitment. Bioact. Mater..

[126] Li C., Wang J., Niu Y., Zhang H., Ouyang H., Zhang G., Fu Y. (2023). Baicalin Nanocomplexes with an In Situ-Forming Biomimetic Gel Implant for Repair of Calvarial Bone Defects via Localized Sclerostin Inhibition. ACS Appl. Mater. Interfaces.

